# Telomere shortening in head and neck cancer: association between DNA demethylation and survival

**DOI:** 10.7150/jca.54760

**Published:** 2021-02-22

**Authors:** Satoshi Yamada, Kiyoshi Misawa, Masato Mima, Atsushi Imai, Daiki Mochizuki, Taiki Yamada, Daichi Shinmura, Junya Kita, Ryuji Ishikawa, Yuki Yamaguchi, Yuki Misawa, Hideya Kawasaki, Hiroyuki Mineta

**Affiliations:** 1 Department of Otolaryngology/Head and Neck Surgery, Hamamatsu University School of Medicine, Hamamatsu, Japan.; 2 Preeminent Medical Photonics Education and Research Center Institute for NanoSuit Research, Hamamatsu University School of Medicine, Hamamatsu, Japan.

**Keywords:** Telomere lengths, Q-PCR, 5-hmC, TET, HNSCC

## Abstract

A growing body of evidence indicates that telomere dysfunction is a biological marker of progression in several types of cancer. However, the association between head and neck squamous cell carcinoma (HNSCC) and telomere length (TL) remains unknown. We measured the absolute TL levels in a well-characterised dataset of 211 tumoral vs normal tissues obtained from the same patients by quantitative polymerase chain reaction assay. Normalised TL levels were significantly lower in tumour samples than in normal tissue (*P* < 0.001) and there was a positive correlation between tumour tissue and normal mucosal tissue (R^2^ = 0.176, *P* < 0.001). We were able to distinguish two classes, one with a tumour/normal TL ratio ≤ 0.3 (38.4%), which showed clear telomere erosion, and the other with a tumour/normal TL ratio > 0.3 (61.6%), in which the TL was slightly shorter or longer than that in normal tissue. Notably, the tumour/normal TL ratio was correlated with the likelihood of disease recurrence (*P* = 0.002), the 5-hydroxymethylcytosine level (*P* = 0.043), and expression of the ten-eleven translocation (*TET*) gene (*P* = 0.043). Our findings show that TL shortening and subsequent low levels of 5-hydroxymethylcytosine and* TET* expression may contribute to development of HNSCC.

## Introduction

Cancers of the upper aerodigestive tract are heterogeneous neoplasms that are treated differently according to their location and whether or not lymphatic dissemination has occurred 1. Despite aggressive multimodal treatment, the survival of patients with head and neck squamous cell carcinoma (HNSCC) remains poor. Although many patients with comparable disease respond similarly to treatment, some ultimately fare much worse or better than expected 2. Therefore, there is a need for a biomarker that can predict the prognosis of the disease, risk of recurrence, and the potential of an identified target to respond to therapy 3. HNSCC is directly or indirectly caused by environmental factors, predominantly smoking (active or passive) and alcohol exposure, so is multifactorial 4. However, apart from molecular studies that can now distinguish human papilloma virus-positive HNSCC, no validated molecular characterisation method has been established 5-8.

Telomeres are located on the ends of each chromosome and contain thousands of tandem repeat sequences of 5'-TTAGGG-3' 9. Telomere length (TL) has been investigated as a cellular biomarker of ageing in response to environmental exposures and lifestyle factors 10. Interest in TL as a potential biomarker of malignancy has grown rapidly, and TL has now been measured in both tumours and normal tissues 11. A polymerase chain reaction (PCR)-based method for determination of TL was considered impossible until the advent of the Cawthon telomere assay, in which there are two targets for amplification: the telomere sequence and the non-variable copy number gene sequence 12.

Previous reports have shown that telomere attrition (shortening) is a frequent event in HNSCC 13, 14. However, although several studies have analysed peripheral blood lymphocytes in patients with HNSCC, few have compared TL in tumour tissue with that in the normal mucosa in the same samples 15. Recent studies have shown that the ten-eleven translocation (TET) enzymes and 5-hydroxymethylcytosine (5-hmC) play essential roles in ensuring the integrity of the genome 16. TET enzymes are important in regulation of telomeres and maintenance of chromosomal stability 17. Our previous research indicates that *TET* mRNA is downregulated in HNSCC as a result of DNA methylation; this may be a critical event in development of HNSCC 18. Furthermore, 5-hmC levels were found to be significantly correlated with relative mRNA levels of *TET* genes 19.

In this study, we measured TL in 211 HNSCC tumours and in the adjacent normal mucosa using PCR analysis. To the best of our knowledge, this is the first report to show a relation between the tumour TL and normal TL in HNSCC. To test our hypothesis, we determined the tumour/normal TL ratio at diagnosis and during follow-up to assess its clinical significance and potential as a prognostic marker for tumour recurrence and patient survival.

## Methods

### Tumour samples

In total, 211 matched pairs of primary HNSCC samples and normal tissue samples were obtained from patients who underwent surgery at the Department of Otolaryngology, Hamamatsu University School of Medicine. The primary tumours were in the oral cavity (n = 61), hypopharynx (n = 66), larynx (n = 52), and oropharynx (n = 32). We obtained tumour tissues and adjacent normal tissues from October 2005 to September 2016. The study protocol was approved by the Institutional Review Board of Hamamatsu University School of Medicine (approval number, 25-149). All patients provided written informed consent. Medical information including patient age and sex, alcohol exposure, smoking status, human papilloma virus status, tumour size, lymph node status, disease stage, and recurrence was collected from patient records.

### DNA extraction and TL measurements

Genomic DNA was extracted from the primary tumours and noncancerous mucosa using a QIAamp DNA Mini Kit (Qiagen, Hilden, Germany). TL was measured using a quantitative (Q)-PCR assay kit (ScienCell Research Laboratories, Carlsbad, CA, USA). Genomic DNA (10 ng) was amplified with a TB Green Premix Ex Taq II system (Takara, Tokyo, Japan) using a Takara Thermal Cycler Dice Real Time System TP8000. The data analysis was conducted according to the manufacturer's instructions. For each DNA sample, two consecutive reactions were performed: the first to amplify a single-copy reference (SCR) gene and the second for the telomere sequence. The SCR primer set recognises and amplifies a 100 bp-long region on human chromosome 17 and serves as a reference for data normalisation. The Q-PCR conditions were as follows: 95 °C for 10 min followed by 32 cycles of 95 °C for 20 s, 52 °C for 20 s, and 72 °C for 45 s. All reactions were performed in triplicate. After Q-PCR was performed, we used the instrument's analysis software to analyse the data 12.

### Quantification of 5-hmC

The 5-hmC content of the primary DNA samples and matched paired normal samples was determined by colorimetric enzyme-linked immunosorbent assay with a Quest 5-hmC DNA ELISA Kit (Zymo Research, Irvine, CA, USA). The assays were performed using 4 μg/mL anti-5-hmC polyclonal antibodies with loading of 200 ng of DNA per well in 178 matched paired samples. Absorbance at 430 nm was evaluated using a SynergyH1 microplate reader and Gen5 software (BioTek, Winooski, VT, USA) according to the manufacturer's instructions. The amount of 5-hmC was calculated as a percentage based on a standard curve generated using kit controls 19. We also calculated the correlation between the ratio of the 5-hmC level in the tumour samples and that in the normal tissue samples.

### RNA extraction and quantitative RT-PCR for TET genes

Total RNA for 123 patients was isolated using an RNeasy Plus Mini Kit (Qiagen). Complementary DNA was synthesised using a ReverTra Ace qPCR RT Kit (Toyobo, Tokyo, Japan). The mRNA levels of *TET1*, *TET2*, *TET3,* and glyceraldehyde 3-phosphate dehydrogenase (*GAPDH*) were measured by quantitative real-time PCR using TB Green Premix Ex Taq II (Takara). The data were analysed using the ΔΔCt method. For each TET gene, the ratio of the expression level between tumour samples and normal samples was calculated. The primer sequences used in this study are shown in Table S1.

### Statistical analysis

The TL and patient data were compared between the matched pairs of tumour and normal tissues using receiver-operating characteristic (ROC) curve analysis. Disease-free survival was measured from the date of the initial treatment to the date of diagnosis of recurrence of disease. Kaplan-Meier tests were used to calculate the probability of survival and log-rank tests to compare survival rates. The prognostic value of methylation status was assessed by multivariate Cox proportional hazards analysis with adjustment for age (≥ 65 years vs. < 65 years), sex, smoking status, alcohol exposure, and tumour stage (I, II, and III vs. IV). A p-value of ≤0.05 was considered statistically significant. The statistical analyses were performed using StatMate IV software (ATMS Co. Ltd., Tokyo, Japan) and the Stata/SE 13.0 system (StataCorp LLC, College Station, TX, USA).

## Results

### TL levels in HNSCC and matched normal mucosa

**Figure 1:**
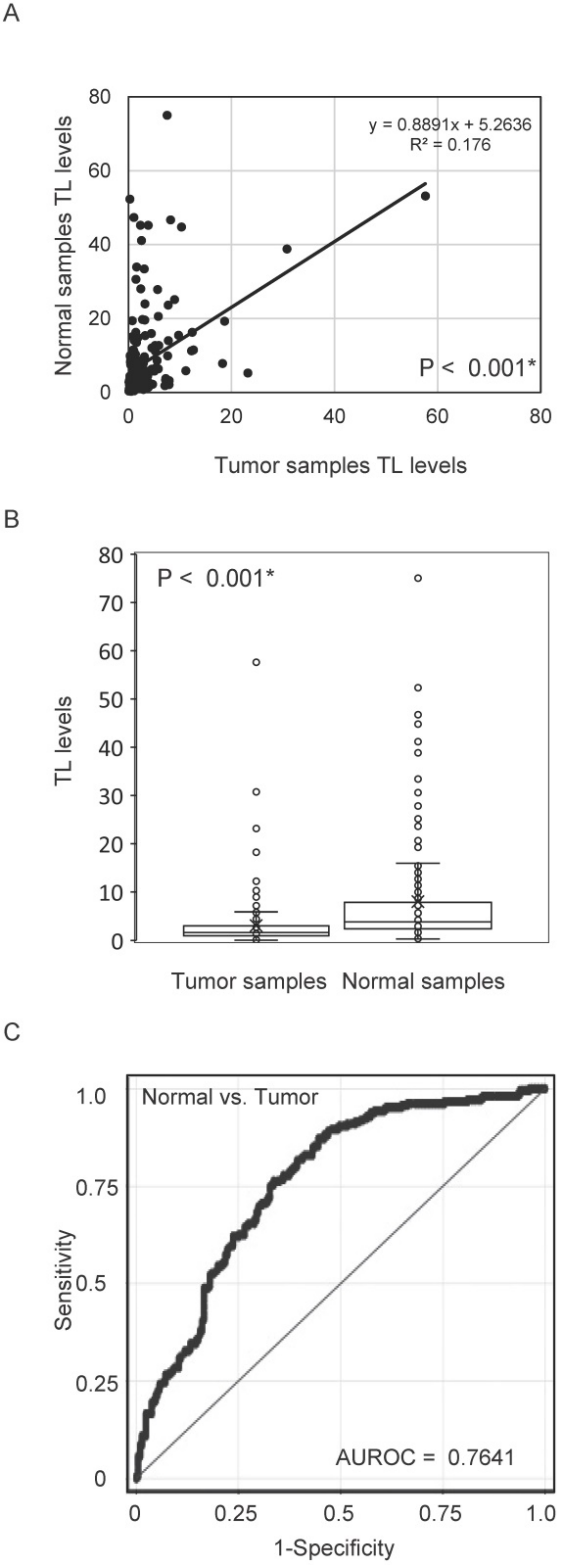
** TL levels in matched pairs of HNSCC tissues and normal mucosal tissues.** (A) Relative telomere length (TL) levels assessed by quantitative polymerase chain reaction. Spearman rank correlations between 211 matched pairs of HNSCC and normal mucosa specimens (*P* < 0.001). (B) Differences between cancerous and normal mucosal tissues were considered significant, as determined by the Student's *t*-test (*P* < 0.001). (C) The AUROC for the TL level was 0.7641. At the cut-off value of 2.370, the sensitivity was 76.3% and the specificity was 66.4%. *P < 0.001.

First, we examined TL DNA levels in 211 matched pairs of tumour tissue and normal mucosa using quantitative PCR. Pearson's correlation analysis revealed a significant positive correlation between TL DNA levels in tumour tissue and those in the matched normal mucosa (R^2^ = 0.176, *P* < 0.001; Figure 1A). Levels of TL DNA were significantly lower in cancer tissues than in matched normal mucosa (3.07 ± 5.30 vs.7.99 ± 11.23; *P* < 0.001, paired* t*-test; Figure 1B). The ROC curve profiles were highly discriminative, clearly distinguishing HNSCC from normal mucosal tissue (area under the ROC curve, 0.7641). At a cut-off value of 2.370, the sensitivity was 76.3% and the specificity was 66.4% (Figure 1C).

### TL levels in 211 matched pairs of HNSCC and normal mucosal samples and clinicopathology

**Figure 2:**
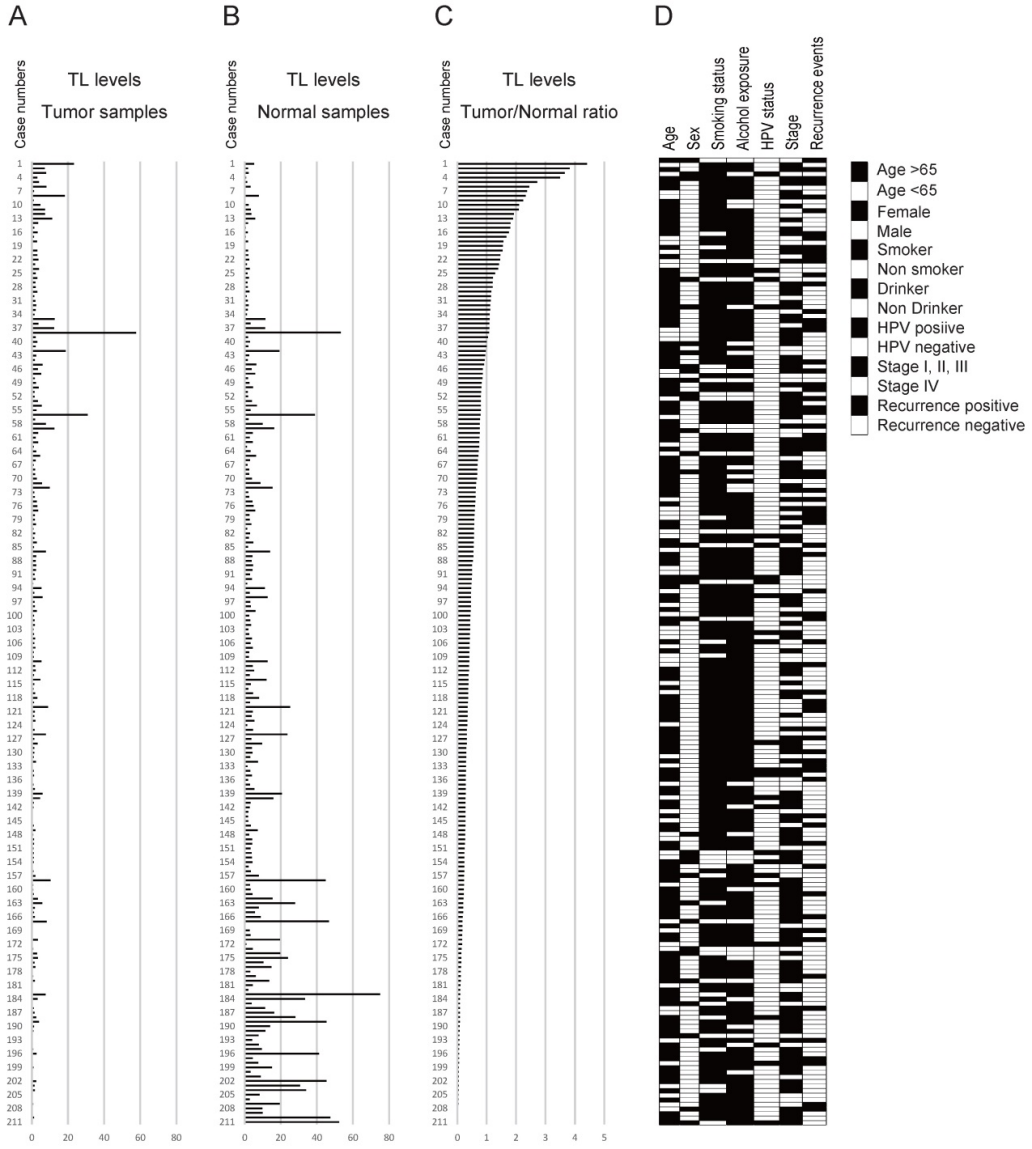
** Diagrammatic representation of the association between TL levels and clinicopathological factors.** Telomere length (TL) levels in (A) tumour and (B) normal samples and (C) the tumour samples/normal samples ratio used for the data are indicated above the figure. The numbers in the left column show the case numbers (labelled 1-211). The bar graph shows the TL levels of the 211 cases. (D) Filled and open boxes indicating clinicopathological discrimination.

The data for the tumour samples, normal mucosa, and the tumour/normal TL ratio are shown in Figure 2A-C. The mean tumour/normal TL ratio was 0.642 (range, 0.005-4.408). The tumour/normal TL ratio was < 1 in 172 cases (81.5%) and ≥ 1 in 39 cases (18.5%; Figure 2C). The clinicopathological classifications are shown in Figure 2D.

**Figure 3:**
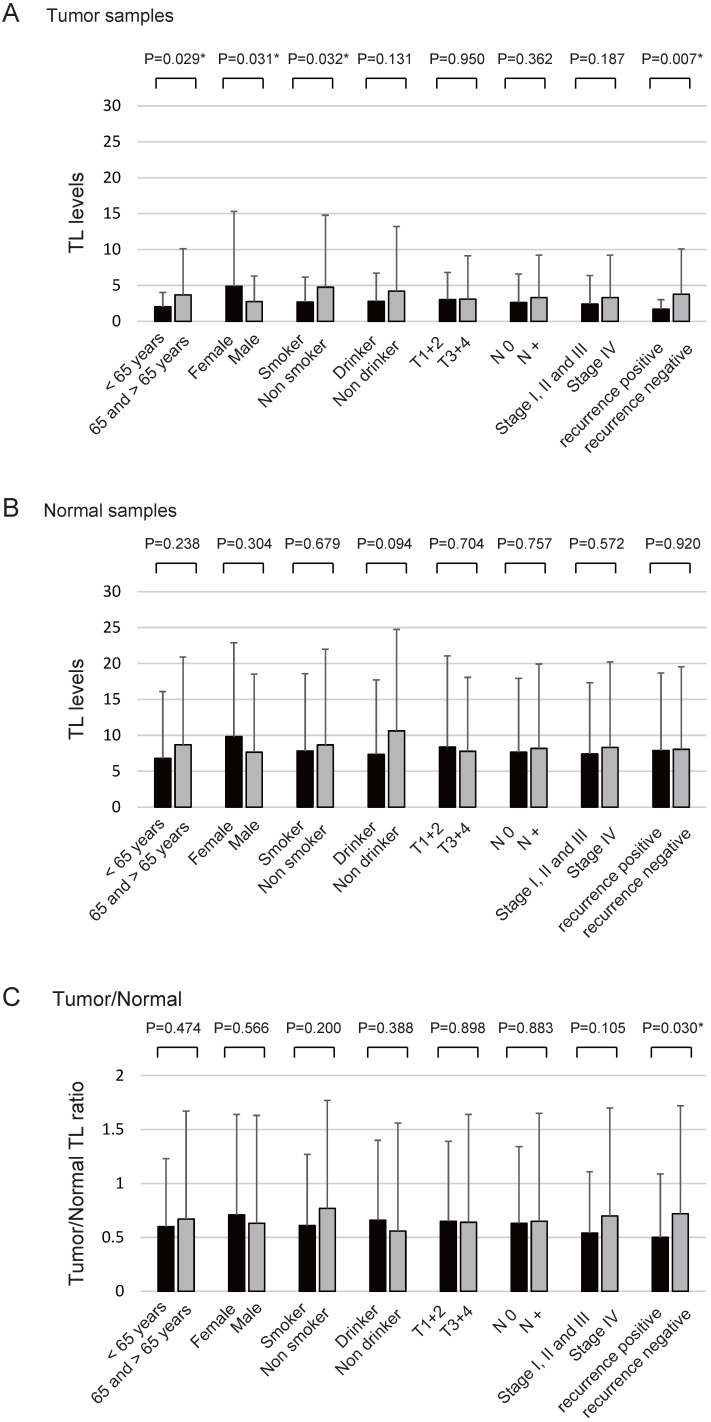
** Association between TL levels and selected clinical parameters.** Telomere length (TL) levels were compared using the Student's *t*-test to determine the association between TL levels and selected epidemiological and clinical characteristics. (A) TL levels of tumour samples: statistically significant differences were found for the associations between TL level and age, sex, smoking status, and recurrence rate. (B) TL levels of normal samples: no differences were noted with regard to any of the clinical characteristics. (C) Tumour/normal TL ratio: statistically significant differences were found for the associations between the TL level and recurrence rate (positive vs. negative). Means and standard deviations are indicated and statistical comparisons between groups are depicted. *P* < 0.05 was considered a statistically significant difference.

The TL levels in the tumour samples were significantly correlated with age (*P* = 0.029), sex (*P* = 0.031), smoking status (*P* = 0.032), and recurrence rate (*P* = 0.007) (Figure 3A) whereas the TL levels in normal samples were not correlated with any clinical parameters (Figure 3B). The tumour/normal TL ratio was significantly correlated with the likelihood of recurrence (*P* = 0.030; Figure 3C).

A positive correlation was found between age of onset of HNSCC and TL levels in the tumour samples (R^2^ = 0.026, *P* = 0.018; Figure S1A). However, in the normal tissue samples, there was no significant correlation of age at disease onset with the TL level or the tumour/normal TL ratio (Figure S1B, S1C).

### Relationship between TL level and patient survival

**Figure 4:**
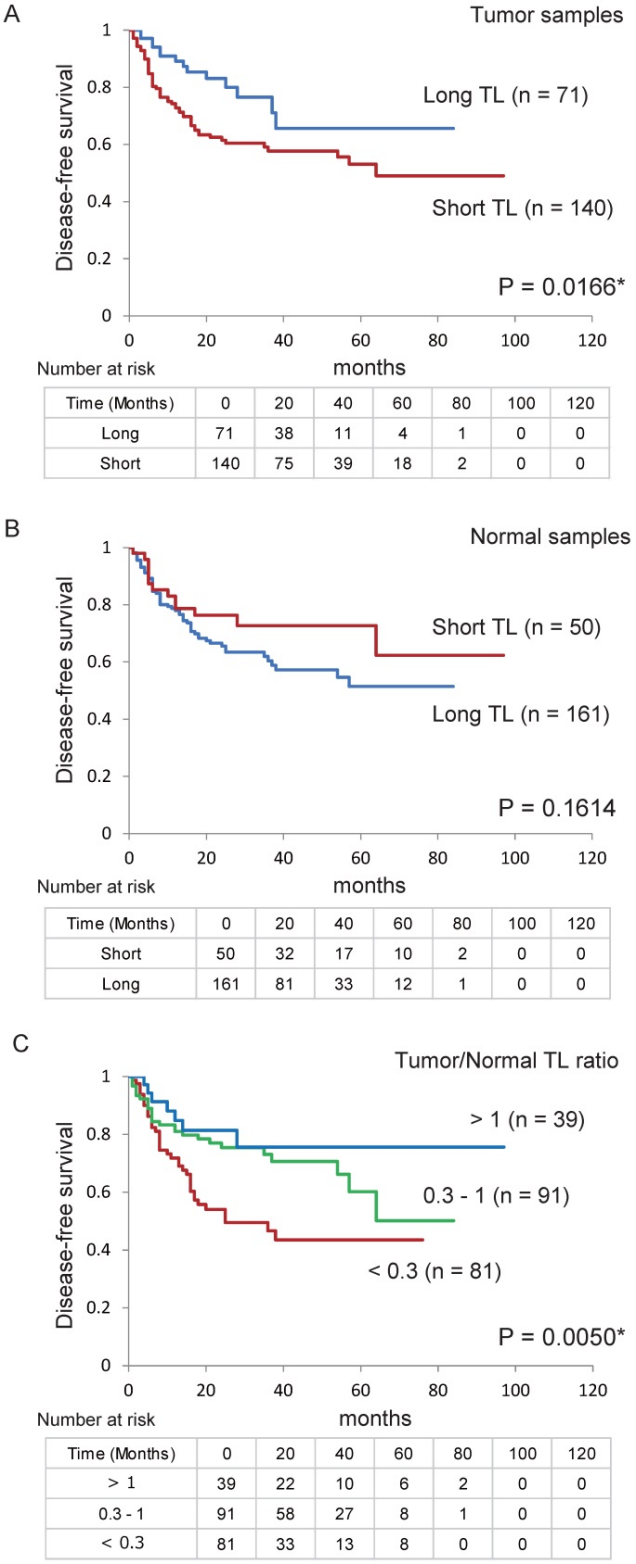
** Kaplan-Meier survival curves based on TL levels in patients with HNSCC.** Disease-free survival according to telomere length (TL) levels in (A) tumour and (B) normal samples. (C) Tumour-to-normal tissue ratio. *P < 0.05.

Next, we confirmed the relationship between DFS in patients with HNSCC and their TL levels using Kaplan-Meier plots (Figure 4). DFS was shorter in patients with a shorter TL (< 2.370) in their tumour sample than in those with a longer TL (> 2.370; *P* = 0.017, log-rank test; Figure 4A). However, there were no relationship between DFS and TL in the normal samples (Figure 4B). Log-rank tests revealed an association between poorer survival and a tumour/normal TL ratio < 0.3 (*P* = 0.002; Table S2). Furthermore, DFS was shorter in the group with a tumour/normal TL ratio ≤ 0.3 than in the group with a ratio of 0.3-1 and the group with a ratio > 1 (*P* = 0.005; Figure 4C).

The association of risk of recurrence with the TL level was estimated using multivariate analysis with Cox proportional hazards models adjusted for age, sex, smoking status, alcohol exposure, and stage. In the tumour samples, the adjusted risk ratio for recurrence was 2.065 (95% confidence interval [CI] 1.134-3.761, *P* = 0.018). In the normal samples, the survival rate was not associated with the TL (adjusted odds ratio for recurrence, 0.576; 95% CI 0.312-1.065; *P* = 0.079). A low tumour/normal TL ratio (<1) was associated with a significantly reduced survival time (hazard ratio, 2.265; 95% CI 1.011-5.076; *P* = 0.047); in the patients with a ratio ≤ 0.3, the adjusted odds ratio for recurrence was 2.425 (95% CI 1.506-3.907; *P* = 0.0003; Table 1).

### Comparison of tumour/normal TL ratio, 5-hmC level, and *TET* expression

The main clinicopathological characteristics are listed in Table 2. When the cut-off value was 0.3, the tumour/normal TL ratio was significantly associated with the likelihood of disease recurrence (*P* = 0.002). No other clinical data were related to the tumour/normal TL ratio. The tumour/normal TL ratio was significantly correlated with the 5-hmC level (high vs. low; *P* = 0.043) and frequency of low expression levels of *TET1*, *TET2*, and *TET3* (0 vs. 1-3; *P* = 0.043; Table 2).

## Discussion

Clarifying the TL level in tumour tissues can provide insights into the mechanisms of tumorigenesis and the risk of disease recurrence for various types of tumours 9. This study found that TL was shorter in head and neck tumour tissues than in the normal mucosa and was associated with a higher risk of recurrence. Our study demonstrates PCR-based tumour/normal TL ratio is associated with a higher risk of early relapse, and is hence, a potential predictive biomarker for HNSCC. Moreover, a low tumour/normal TL ratio was associated with a decreased 5-hmC level and low *TET* gene expression in HNSCC. Elucidation of TL may provide insights into the mechanisms underlying tumorigenesis and the risk of disease recurrence in HNSCC.

The present study aimed to investigate the role of TL as a molecular marker of disease severity. To facilitate high-throughput TL measurements, the quantitative PCR assay remains the most cost-effective method for large-scale epidemiological and population studies 20. This assay is relatively easy to perform and does not require a large amount of starting DNA (approximately 5 ng). TL decreases with ageing and contributes to cell senescence 21. No correlation was found between age and TL in our normal tissue samples. This finding was unexpected because TL in non-cancerous cells is inversely correlated with age. In agreement with other studies, including those in head and neck cancer 13, 14, we found that TL in cancer tissues increased with age and the aggressiveness of the disease. Moreover, the tumour/normal TL ratio did not increase with age but was associated with an increased risk of recurrence. Telomere shortening is associated with colorectal carcinogenesis in both tumour tissues and normal mucosa 22. Patients with colorectal adenoma or carcinoma to clarify the respective contributions of tumour and normal TL to colorectal cancer initiation and progression 23. In hepatocellular carcinoma patients, shortened telomeres in tumour cells and cancer-associated fibroblasts compared with their normal counterparts were independently and significantly associated with the clinical outcome 24. These data support the concept that a declining tumour/normal TL ratio in an advanced carcinoma augments the enhanced replication activity during progression of cancer.

Telomere shortening is an early event that contributes to development of genomic instability, which plays an important role in the initial steps of carcinogenesis 25. Thus, shortening of human telomeres has two opposing effects: tumour suppression by inducing cell death and tumour promotion by causing genetic instability 26, 27. Recently, it was reported that TET enzymes play important roles in maintenance of telomeres and chromosomal instability in mouse embryonic stem cells 16. Loss of telomeres resulting from TET deficiency may have implications in ageing and cancer 17. TET family proteins can convert 5-mC to 5-hmC, which is widely accepted as the sixth base in the mammalian genome, following 5-mC, the fifth base 28. Therefore, low 5-hmC levels are associated with reduced TL 29. Depletion of 5-hmC could contribute significantly to genomic instability and incorrect segregation of chromosomes, perhaps explaining the relationship between low 5-hmC levels and cancer 30. Both 5-hmC and TET expression levels might contribute to genomic instability in various human cancers, including HNSCC.

As a prognostic biomarker, tissue TL outperforms existing clinical risk parameters. Several studies have investigated the relationship between TL and clinical features in squamous cell carcinoma (SCC) 31-33. In cancer tissues, TL is linked to risk factors for oesophageal SCC in consumers of alcohol 31 and to cytologic biomarkers for cervical cancer 32. Furthermore, in non-melanoma skin cancer, TL was found to be significantly lower in SCC than in basal cell carcinoma, Bowen's disease, or keratosis 33.

In summary, we have demonstrated that the tumour/normal TL ratio is abnormally low in patients with a poor prognosis, and this may be a critical event in progression of HNSCC. The current method used to assess risk recurrence in patients with HNSCC is imprecise - indeed, half of such tumours recur after curative surgery. These findings can benefit HNSCC screening and surveillance algorithms. We also found that changes in the 5-hmC level and TET expression levels were correlated with the tumour/normal TL ratio. If these data can be validated in larger series, they may lead to new post-therapeutic surveillance strategies that include more personalised follow-up.

## Supplementary Material

Supplementary figures and tables.

## Figures and Tables

**Table 1:** TL levels associations with disease-free survival using Cox proportional hazards model in 211 patients

Samples	Overall (%)	Recurrence events				Adjusted RR (95% CI)^b^
		Positive (N = 71)	Negative (N = 140)	P^a^	P^b^	
**Tumor samples**						
Short	140(66.4%)	57	83			
Long	71 (33.6%)	14	57	0.002*	0.018*	2.065 (1.134-3.761)*
**Normal samples**						
Short	50 (23.7%)	13	37			
Long	161(76.3%)	58	103	0.231	0.079	0.576 (0.312-1.065)
**Tumor/Normal ratio**						
≤ 1	172(81.5%)	64	108			
> 1	39 (18.5%)	7	32	0.024*	0.047*	2.265 (1.011-5.076)*
**Tumor/Normal ratio**						
≤ 0.3	81 (38.4%)	38	43			
> 0.3	130(61.6%)	33	97	0.002*	0.0003*	2.425 (1.506-3.907)*

a: Fisher's exact probability test. b: Adjusted for age, sex, smoking status, alcohol exposure and stage. * P<0.05.

**Table 2:** The correlation between TL levels and clinical characteristics, 5hmC levels and TET genes expression

Samples			TN TL ratio		
Characteristics		Overall (%)	< 0.3	> 0.3	*P*
Age	≤ 65	78 (37.0%)	27	51	
	> 65	133 (63.0%)	54	79	0.464
Sex	female	33 (15.6%)	15	18	
	male	178 (84.4%)	66	112	1
Smoking status	smoker	173 (82.0%)	67	106	
	non smoker	38 (18.0%)	14	24	0.856
Alcohol exposure	drinker	170 (80.6%)	61	109	
	non drinker	41 (19.4%)	20	21	1
Tumor size	T1-2	78 (37.0%)	29	49	
	T3-4	133 (63.0%)	52	81	0.884
Lympho-node status	N0	75 (35.5%)	28	47	
	N+	136 (64.5%)	53	83	0.883
Stage	I, II, III	75 (35.5%)	31	44	
	IV	136 (64.5%)	50	86	1
Recurrence events	positive	71 (33.6%)	38	33	
	negative	140 (66.4%)	43	97	0.002*
TN 5hmC ratio	high	34 (19.1 %)	14	38	
	low	144 (80.9 %)	55	71	0.043*
TN TET1 expression ratio	high	66 (60.0 %)	20	46	
	low	44 (40.0 %)	18	26	1
TN TET2 expression ratio	high	66 (60.0 %)	22	44	
	low	44 (40.0 %)	16	28	1
TN TET3 expression ratio	high	64 (58.2 %)	19	45	
	low	46 (41.8 %)	19	27	0.228
Frequency of TET1, TET2 and TET3 low expression	0	47 (42.7%)	11	36	
	1-3	63 (57.3%)	27	36	0.043*

† Fisher's exact probability test. * P<0.05.
