# Supplementary material for: Telomere shortening in head and neck cancer: association between DNA demethylation and survival
Source: J Cancer. 2021 Feb 22;12(8):2165–72. doi: 10.7150/jca.54760 (PMC7974875; doi:10.7150/jca.54760)
Supplement: Supplementary file 1 — Supplementary figures and tables. [file jcav12p2165s1.pdf]

Figure S1 Kiyoshi Misawa

A

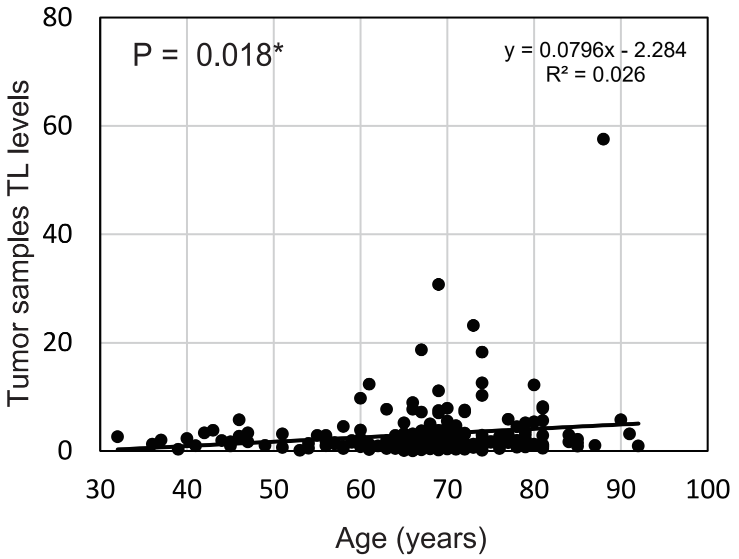

B

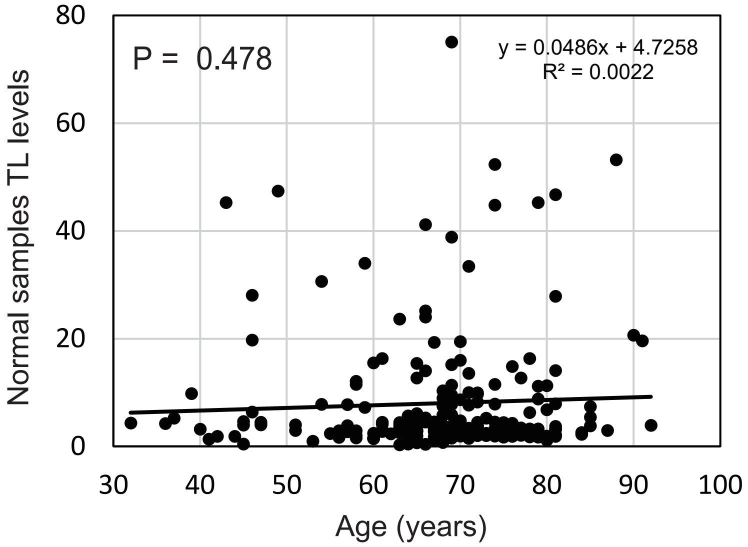

C

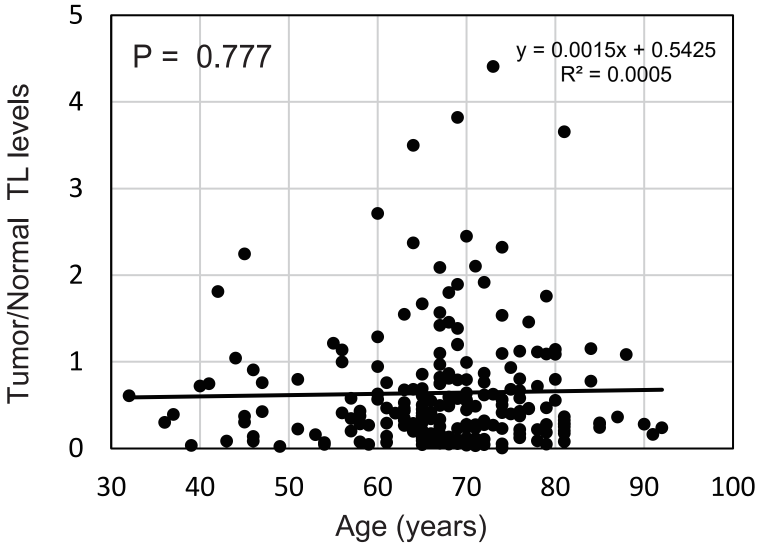

Table S1. Q-RT-MSP Primer List

Gene	Forward primer 5’-3’	Reverse primer 5’-3’
GAPDH	GCACCGTCAAGGCTGAGAAC	TGGTGAAGACGCCAGTCTCTA
TET1	CCCTTGGAAATGCCATAGGAA	GAGAGCCTGCTGGAACTGTTG
TET2	GGCTGTTGGCCAGAGACTTA	ATACCTGTAGGTGTTTGCCTGTTTA
TET3	GCCAACTTCAACATACCCTGGAC	CACCTGGATGTGGGACTGTGTAA

Table S2. Results of log-rank tests for effect of cut off of tumor/normal TL ratio ratio on disease free survival in 211 HNSCC.

Cut off of TN TL ratio	No. patients with profile	P †
<1	172	0.0654
<0.9	166	0.0742
<0.8	159	0.057
<0.7	146	0.013
<0.6	135	0.010*
<0.5	121	0.032*
<0.4	101	0.012*
<0.3	81	0.002*
<0.2	48	0.085
<0.1	29	0.008*

† Log-rank test
